# Illuminating Progress: The Contribution of Bioluminescence to Sustainable Development Goal 6—Clean Water and Sanitation—Of the United Nations 2030 Agenda

**DOI:** 10.3390/s23167244

**Published:** 2023-08-18

**Authors:** Denise Gregucci, Faisal Nazir, Maria Maddalena Calabretta, Elisa Michelini

**Affiliations:** 1 Department of Chemistry “Giacomo Ciamician”, University of Bologna, Via Selmi 2, 40126 Bologna, Italy; denise.gregucci2@unibo.it (D.G.); faisal.nazir2@unibo.it (F.N.); maria.calabretta2@unibo.it (M.M.C.); 2 Center for Applied Biomedical Research (CRBA), Azienda Ospedaliero-Universitaria Policlinico S. Orsola-Malpighi, 40138 Bologna, Italy; 3 Health Sciences and Technologies Interdepartmental Center for Industrial Research (HSTICIR), University of Bologna, 40126 Bologna, Italy

**Keywords:** bioluminescence, biosensors, bioassays, water quality monitoring, Sustainable Development Goal 6, water pollution

## Abstract

The United Nations Agenda 2030 Sustainable Development Goal 6 (SDG 6) aims at ensuring the availability and sustainable management of water and sanitation. The routine monitoring of water contaminants requires accurate and rapid analytical techniques. Laboratory analyses and conventional methods of field sampling still require considerable labor and time with highly trained personnel and transport to a central facility with sophisticated equipment, which renders routine monitoring cumbersome, time-consuming, and costly. Moreover, these methods do not provide information about the actual toxicity of water, which is crucial for characterizing complex samples, such as urban wastewater and stormwater runoff. The unique properties of bioluminescence (BL) offer innovative approaches for developing advanced tools and technologies for holistic water monitoring. BL biosensors offer a promising solution by combining the natural BL phenomenon with cutting-edge technologies. This review provides an overview of the recent advances and significant contributions of BL to SDG 6, focusing attention on the potential use of the BL-based sensing platforms for advancing water management practices, protecting ecosystems, and ensuring the well-being of communities.

## 1. Introduction

The 2030 Agenda for Sustainable Development was adopted by the United Nations Member States in 2015 to provide both a guide and an action plan for peace and prosperity through the achievement of 17 Sustainable Development Goals (SDGs). In particular, SDG 6 aims at ensuring the availability and sustainable management of water and sanitation for all [1]. To achieve this goal, innovative approaches for the sustainable management of water resources are required, contributing to improving environmental sustainability, human health, and economic development as well.

Water is an essential resource for human survival, and it plays a critical role in the functioning of ecosystems. Its unique properties make it indispensable in various chemical, biological, and environmental processes. Organic carbon, nutrients (such as nitrogen and phosphorus), and metals (such as potassium, copper, and silver) are typically abundant in waste streams that can be recovered in agreement with circular economy principles [2]. At the same time, heavy metals, organic matter, personal care products and pharmaceutical residues, plastics, pesticides, and pathogens are among the pollutants of major concern [3]. Several efforts have been made to provide regulations and directives for ensuring water quality such as the EU Framework Directive (2000) [4], the Urban Wastewater Treatment Directive (under revision) [5], and the US Clean Water Act and US Safe Drinking Water Act [6]. 

**Figure 1:**
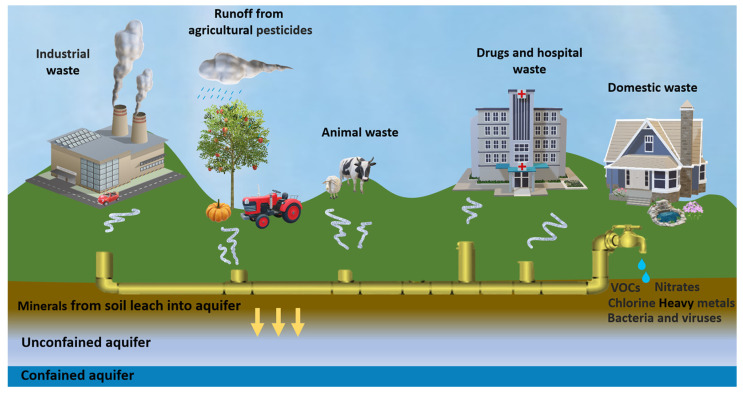
Schematic representation illustrating potential sources of groundwater contamination.

Water quality is also affected by the presence of microbiological contaminants in drinking and tap water, such as bacteria, viruses, and protozoa, causing serious threats to the entire water ecosystem. Monitoring waste streams, optimizing the production process, detecting contaminants and pollutants, and recovering useful compounds [7] can contribute to fulfilling the concept of the 4Rs (reduce, reuse, recycle, and recover) of the circular economy [8]. In light of the definition of the circular economy by the European Union Commission [9], innovative approaches are necessary to reuse water and recover valuable materials [10,11]. Novel strategies are thus required to face water scarcity and ensure water security, also considering that by 2050 the urban water demand is expected to increase by 80%, and only two cities, Amsterdam and Singapore, were classified as “water-wise cities” [12]. Monitoring the aquatic environment and applying efficient methods for its protection is impossible without employing adequate chemical analytical methods [13]. Ideal techniques should be cost-effective, environmentally friendly, selective, and sensitive enough to detect analytes in traces with the required precision and accuracy, even on-site and at the most critical points of the water cycle (Figure 1). 

The routine monitoring of water contaminants requires accurate and fast response detection techniques. Laboratory analyses and conventional methods of field sampling still require considerable labor and time with highly trained personnel and transport to a central facility with sophisticated equipment, which renders routine monitoring cumbersome, time-consuming, and costly. Thanks to their high selectivity and sensitivity, standard analytical methods, such as mass spectrometry and high-performance liquid chromatography, have gained significant popularity in the detection and identification of toxic compounds in environmental matrices. However, these methods do not provide information about the actual bioactivity, and potential toxicity of water and environmental samples, which is crucial for characterizing complex samples, such as urban wastewater and stormwater runoff.

Instead, biosensors, relying on biological recognition elements can provide relevant toxicological information and, most notably, can be deployed on-site, thus being suitable for real-time point-of-need analyses [14,15]. Several sensors and biosensors have been proposed relying on different detections, including portable X-ray fluorescence (PXRF) spectrometry, which offers the possibility to perform non-destructive analysis on both liquid and solid samples [16]. Electrochemical techniques, such as potentiometric, amperometric, voltammetric, and coulometric methods, are widely used because they are simple, cost-effective, and fast [17]. Spectroscopic analytical techniques, such as flame or electrothermal atomization atomic absorption, X-ray fluorescence, inductively coupled plasma optical emission spectroscopy, neutron activation analysis, ion chromatography ultraviolet-visible, total reflection X-ray fluorescence, laser-induced breakdown, and atomic fluorescence are straightforward and generally very sensitive [18]. They are characterized by very low limits of detection (LODs) and can detect a wide range of heavy metals simultaneously [19,20]. Inductively coupled plasma mass spectrometry (ICP-MS) is widely used for analyzing heavy metals in water samples. However, it requires expensive equipment and skilled personnel. The benefits of ICP-MS include the capability to analyze multiple elements at once and having extremely low LODs, which is crucial for assessing potential adverse health effects [21]. It has been successfully used to directly measure heavy metals in seawater, even with significant matrix effects. Ongoing research is also focused on coupling ICP-MS with automated flow-injection systems, showing promise as a practical analytical approach. However, all these approaches are still based on complex and time-consuming laboratory procedures, including sampling, pretreatment, and expensive instrumentation. In addition, they require highly skilled personnel. For this reason, there is a significant interest in the advancement of sensors and biosensors for on-site water monitoring. Such technology would not only allow for more affordable analysis but also real-time tracking of target pollutants and contaminants. This would lead to both a significant cost reduction and an enhancement of the sustainability of the overall chemical analysis procedure. The availability of such techniques, in compliance with the principles of Green Analytical Chemistry principles [22] and the more comprehensive White Analytical Chemistry principles [23], would surely provide a great contribution to the achievement of SDG 6.

## 2. Bioluminescence Tools

**Figure 2:**
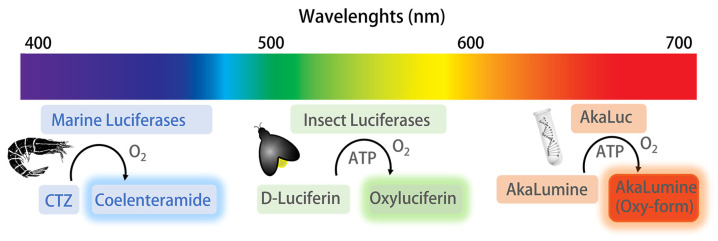
BL luciferase–luciferin pair systems obtained from marine and terrestrial organisms, and from engineered proteins. The BL reaction from marine organisms involves the oxidation of coelenterazine (CTZ) substrate by luciferase; CTZ is converted to the coelenteramide form, emitting photons in the blue light region (454–493 nm). BL reactions from insects involve the oxidation of D-luciferin in two distinct steps, in the presence of ATP, to obtain the oxyluciferin form, emitting photons in the green-red region of the visible spectrum. The AkaLuc system involves the use of an engineered firefly luciferase AkaLuc optimized to catalyze the oxidation of unnatural luciferin AkaLumine substrate to obtain the AkaLumine oxy-form, emitting photons in the near-infrared region.

Bioluminescence (BL) is defined as the emission of light from a natural chemical process in various terrestrial and marine living organisms, such as bacteria, fish, insects, mollusks, and fungi [24,25]. A BL reaction involves BL proteins, called luciferases, that catalyze a multi-step oxidation of the substrate luciferin (Figure 2). Thanks to synthetic biology and organic chemistry tools, up to now a wide portfolio of luciferase–luciferin systems is available, providing enhanced or shifted emissions such as the AkaLumine–AkaLuc and the Nanoluc–furimazine systems [26,27].

One of the most studied luciferase–luciferin systems involves beetle luciferases, which require the substrate D-luciferin (D-LH_2_) and specific co-factors (ATP and Mg^2+^) [28]. This BL mechanism involves the formation of luciferyl-adenylate (LH_2_-AMP) followed by a reaction with molecular oxygen with the production of an oxidated electronically excited state, which rapidly decays to the ground state through a radiative relaxation, and emits photons. As a common feature of chemiluminescence, no light source is required in BL, which is characterized by a high signal-to-noise ratio, it exhibits efficient quantum emission yields, and the main issues related to fluorescence detection, such as photobleaching and autofluorescence from compounds, media, or cells, are avoided [29,30]. 

The quantum yield (ϕ_BL_) of the emission depends on the luciferin structure and on the aminoacidic sequence of the luciferase active site; for the wild-type North American firefly *Photinus pyralis* Luc (PpyLuc) luciferase reaction, the ϕ_BL_ is about 45% [28,31,32]. 

This turns out to be a great advantage for the detection of samples with a very low concentration of analytes or in cases where it is necessary to have miniaturized systems for developing lab-on-a-chip platforms for on-site analysis. Nowadays, the requirement of adenosine triphosphate (ATP) as a cofactor has made luciferases a valuable tool in each application in which ATP detection is required, e.g., quantification of intracellular ATP levels or for hygiene monitoring [33,34,35]. BL proteins, including both luciferases and photoproteins, are indeed commonly used as reporters and labels in immunoassays, intracellular ATP or Ca^2+^ detection, gene expression, DNA and RNA hybridization assays, bioluminescence resonance energy transfer (BRET) assays, and whole-cell biosensors [36,37,38].

A variety of marine organisms also exhibit BL, but only a small number of bioluminescent proteins derived from these organisms have been employed in analytical applications. The production of light in marine bacterial luciferases involves a two-step mechanism, in which luciferase bounded to the reduced flavin mononucleotide and molecular oxygen produces a peroxyflavin intermediate. Secondly, this intermediate reacts with the bacteria’s endogenous aldehyde luciferin to form a second intermediate, which rapidly decays, emitting light at a specific wavelength [39]. This system does not require any external substrate. In contrast with other organisms that have separate genes controlling the luciferase enzyme synthesis and the luciferin-synthesizing enzymes, the lux cassette gene (luxCDABE) of bacterial luciferases encodes both the luciferase enzyme and enzymes responsible for synthesizing the necessary substrates for the BL reaction. The implementation of lux cassette as a reporter gene in biosensor applications provides the non-negligible advantage of avoiding the need for external substrate addition. In this sense, numerous biosensing systems based on whole cells and proteins have been developed and used to monitor various parameters such as water toxicity, the presence of heavy metals, quorum-sensing molecules, and organic compounds [40,41,42,43]. 

Among insects’ luciferases, PpyLuc is the most investigated luciferase. PpyLuc is a 62 kDa protein characterized by yellow-green light emission (λ_max_ 562 nm) due to its reaction with the D-luciferin substrate [44]. Blue-emitting luciferases were also explored. They were isolated from marine species, and they react with CTZ substrate instead of luciferin, without requiring any cofactor except for molecular oxygen [45,46].

Since wild-type luciferases are generally heat-sensitive enzymes, which rapidly lose the catalytic activity at 37 °C, several efforts were aimed at improving BL enzymes’ stability and emission properties [47]. Starting from the pioneering work of White et al. [48], several strategies have been exploited to improve luciferase stability at higher temperatures and at different pH levels, using site-directed and random mutagenesis. A modified chimeric enzyme that combines the N-domain of PpyLuc with the C-domain of *Luciola italica* luciferase was also obtained [32]. This new luciferase showed an activity enhancement of 90% and a stabilization at high temperatures and at a low pH. Recently, a small (13.9 kDa) thermostable luciferase characterized by a melting temperature higher than 95 °C has been explored by a de novo enzyme approach [49]. Several luciferase mutants and D-luciferin or CTZ analogs were also obtained in order to have a broad range of emission wavelengths from blue to green and from orange to red [50,51]. Cells can be genetically modified to produce bioluminescent reporters under the regulation of constitutive promoters or inducible transcription elements to react to various physical and biochemical stimuli. 

The current goal is to create luciferases with the following characteristics: small size, high stability, good expression in living cells, specificity for one substrate, and pH-insensitive emission. On the other hand, research is focused on the development of highly sensitive photodetectors and on the implementation of BL into low-cost portable sensing systems. 

BL is considered a well-established optical detection platform, widely exploited in bioanalytical applications. Compared to absorbance and fluorescence detection techniques, BL generally offers a wider dynamic range and a higher sensitivity due to lower background interferences, and no external excitation light source is required. In addition, homogeneous BL assays can be developed with straightforward protocols, enabling easy automation, miniaturization, and suitability for high-throughput applications [30]. 

By combining the natural BL phenomenon with cutting-edge technology, BL biosensors offer a promising solution for all applications in which sensitive yet low-cost (average cost per assay EUR 0.2–5.0) monitoring of target analytes is required.

Biosensors, comprising a biological recognition element and a transducer, provide qualitative, quantitative, or semi-quantitative analytical information, recreating a miniaturized laboratory in an all-in-one device, such as portable analytical devices (PADs) and lab-on-a-chip systems for point-of-care and point-of-need applications, and are capable of rapidly detecting analytes with high accuracy [52].

In addition, BL-based sensing platforms are non-destructive and non-invasive, making them suitable for real-time monitoring and longitudinal studies. 

Biosensors have the potential to play a crucial and multifaceted role in achieving the SDGs. For instance, biosensors can contribute directly to SDG 6 by monitoring the quality of water in real time, detecting pollutants, and ensuring compliance with environmental regulations. Biosensors that are reusable and consume minimal energy align with SDG 12, promoting responsible consumption and production practices. Moreover, biosensors can also play an essential role in monitoring climate change and contributing to SDG 13. Additionally, they can play a role in protecting the environment, in line with SDG 14 [53,54].

## 3. BL Biosensors Applied to Water Quality Monitoring

Over the past 40 years, biosensors have emerged as promising tools for the rapid detection of pollutants in several matrices. According to the Point-Of-Care Testing (POCT) concept, tests should be performed on-site with handheld devices, fulfilling the “REASSURED” requirements: Real-time connectivity, Environmental friendliness, Affordable, Sensitivity, Specificity, User-friendly, Rapid, Equipment-free, and Deliverable [55]. Such devices offer a cost-effective alternative to costly and time-consuming laboratory tests. Several kinds of biosensors have been used for the determination of pollutants in water samples, including optical-based biosensors [56,57,58], showing relatively high sensitivity with the advantage of providing real-time qualitative and quantitative analysis without extensive sample preparation. Although less explored, BL-based biosensors could provide more advantages, especially in terms of sensitivity and compactness of the device, without needing an energy supply or light sources such as fluorescent and electrochemical sensors [59]. 

### 3.1. BL Biosensors for Water Pollutants

Probably the most widespread application of BL is the use of luciferase–luciferin reactions to monitor microbial contamination via ATP detection. User-friendly, ready-to-use and stable ATP sensing paper biosensors that can be combined either with a smartphone or with sensitive portable photodetectors have been developed [33,35]. As an example, we reported a smartphone-based paper sensor enabling the detection of ATP down to 10^−14^ mol with a cost per sample of about EUR 0.5, thus 5–10 times lower than commercial assays [34]. However, mixtures of contaminants, including chemical or microbial substances, can be generally present in water bodies; therefore, more holistic approaches are required to try to detect as many threats as possible. In this context, the use of genetically modified microbes able to respond to different classes of contaminants producing a proportional BL signal is highly valuable. In addition, these bacteria can be easily immobilized and integrated into portable devices. 

Denisov et al. developed a luciferase-based disposable microfluidic chip for water pollution testing [60]. Luciferase enzyme from *Photobacterium leiognathi* and the NAD(P)H:FMN-oxidoreductase from *Aliivibrio fischeri* were immobilized in the microfluidic chip and the BL reactions activated through sample addition. The proposed system was evaluated using copper (II) sulfate, 1,3-dihydroxybenzene, and 1,4-benzoquinone as model compounds, obtaining LODs of 3 μM, 15 mM, and 2 μM, respectively. These results were in agreement with those obtained with conventional environmental biosensors based on freeze-dried bacteria. The microfluidic chip stability after long-term storage was also evaluated at different temperatures, showing good responsiveness to pollutants for four months at +4 °C and for three weeks at room temperature. Several BL assays to detect potential toxic compounds with inhibitory effects on bacterial BL systems have been developed [61,62]. The proposed biosensors exploited the BL bacterial coupled enzyme system NAD(P)H:FMN-oxidoreductase-luciferase for environmental and food applications and a six sequential stage procedure was designed to minimize potential matrix effects on the activity of the BL biosensor.

Recently, Yu et al. developed a biosensor based on bioluminescence resonance energy transfer (BRET), capable of rapidly detecting inorganic phosphate (Pi) in water with high sensitivity and selectivity. NanoLuc luciferase and Venus fluorescent protein were used as the BL energy donor and acceptor, respectively [63]. In particular, the Pi-specific binding protein (PiBP), fused between the BL donor and the fluorescent acceptor, was used as molecular recognition for the Pi causing a conformational change that affects the energy transfer efficiency. Under optimized conditions, a LOD of 1.3 μg L^−1^ and a detection range from 3.3 to 434 μg L^−1^ were obtained. The sensor showed acceptable accuracy when applied to real water samples, thus representing a sensitive, fast, and environmentally friendly alternative to the traditional phosphomolybdenum blue spectrophotometry method for the detection of phosphate in water. 

Microplastics are surely one of the most concerning classes of pollutants among emerging contaminants. Their ubiquity and interaction with other contaminants have been widely reported, although their effects on human and wildlife health still need investigation. The scientific community also proposed to define the new historical era we are living in as the Plasticene [64]. The effect of polystyrene submicron particles on the detection of geno- and cytotoxicity in wastewater treatment plants was assessed with BL genetically modified bioluminescent bacteria. Interestingly, no direct toxicity was observed in bacteria, but a masking effect was observed leading to a reduction in the capability of bacteria to detect toxicities in water samples. Further studies will be required to clarify this behavior [65]. 

A large body of evidence suggests that endocrine disruptors are present in several water bodies, deriving from municipal or industrial wastewater, and international legislation has recognized them as priority substances [65,66]. To detect endocrine disruptors, several yeast bioreporters were developed and integrated into portable devices and also connected to smartphones or action cameras with the great advantage of avoiding the need for additional instrumentation. A very sensitive S. cerevisiae biosensor for estrogenic compounds was reported, providing a LOD of 0.08 nM for 17β-estradiol [67]. The possibility to use smartphones is very important since BL systems do not require an electrical energy supply for the reaction and require very low volumes of green reagents (a few tens of µL), therefore, significantly reducing the cost and the carbon footprint of the sensors. On the other hand, it must be pointed out that more consumables are required when compared to fluorescence-based methods since the substrate luciferin is needed for the BL emission. 

A more holistic approach was used by Bazin et al., who exploited a panel of BL biosensors to assess the quality of water samples. Different bioactivities were evaluated, including oxidative stress (superoxide radical or hydroxyl radical), protein damage, cell membrane damage, and cellular toxicity using five recombinant luminescent Escherichia coli strains harboring plasmids pSodALux, pKatGLux, pGrpELux, pFabALux, and pLITE2, respectively. A yeast strain for estrogenic activity was also used relying on β-galactosidase as the reporter gene [68]. 

To improve the detection of antibacterial compounds, Melamed et al. engineered bacteria by fusing the promoter of the soxS regulator to the Photorhabdus luminescens luxCDABE cassette. Membrane permeability was increased by a dual strategy comprising both overexpression of a porin (OmpF), which enables passive diffusion of molecules, and introduction of mutations in the efflux system. Thanks to these strategies, the authors were able to detect tetracyclines at concentrations (5.4 ng/mL) even lower than those reported in water samples [69].

### 3.2. BL Biosensors for Toxicity Evaluation

Monitoring the toxicity of water is of vital importance to detect pollutants and compounds used for sanitation and purification. Ozone and chlorine are often used for water chemical purification; however, their use is associated with the formation of toxic disinfection byproducts, which cause health risks to humans. Toxic effects of chlorinated and ozonated wastewater effluents were evaluated by Bhuvaneswari et al. thanks to the obtainment of three genetically modified bioluminescent bacteria for cytotoxicity, genotoxicity, and reacting oxygen species (ROS) generation. By comparing results obtained with the naturally isolated cyanobacteria *Spirulina* sp., bioluminescent bacteria showed not only increased sensitivity to toxicity induction but also provided mechanism-specific responses associated with specific stimuli in wastewater effluents [70]. The BL bacterial response to the specific toxicity of the developed mechanism differs among wastewater effluents, reflecting the variability in effluent compositions.

Another main issue is the monitoring of organic pollutants, for example, petroleum hydrocarbon in aquatic environments. Water toxicity monitoring involves the use of bioindicators and biomonitors such as microorganisms, plants, fish invertebrates, and algae. By measuring the biological endpoint of growth, reproduction, or survival in these bioassays, it is possible to obtain toxicity assessment. Rapid, non-invasive, reproducible, and user-friendly bioassays are based on natural bioluminescent bacteria. These bacteria are considered a useful tool for monitoring toxicity since BL represents a sensitive indicator of their metabolic status, which is inhibited by the presence of toxic substances. 

Mirjani et al. developed a BL inhibition bioassay to evaluate the toxicity of total petroleum hydrocarbons (TPHs) in aquatic environments [71]. In this study, natural bioluminescent *Aliivibrio fischeri* bacteria were used, which emit a blue-green light under optimal environmental conditions, particularly in the presence of high amounts of oxygen. Short-term (15 min incubation) and long-term (16 h incubation) toxicity were assessed and no toxicity for various concentrations of TPHs (concentration range from 30 to 220 mg/L) was observed. A half-maximal effective concentration (EC50) of 1.77 mg/L was reported after 6 h of incubation. 

An interesting approach to simultaneously monitor genotoxicity and cytotoxicity was developed by Baumstark-Khan et al., who proposed a Lux-Fluoro bioassay based on bacteria genetically modified to express the lux operon from the bioluminescent marine photobacterium *P. leiognathi* under the control of a DNA damage-dependent promoter and the green fluorescent protein (GFP) from the jellyfish *Aequora victoria* under the control of a constitutive promoter. In the presence of a genotoxic chemical, the inducible promoter is activated leading to the expression of the lux operon, obtaining a BL signal proportional to the concentration of the genotoxic compound. For toxicity assessment, a decreased signal is obtained proportionally to the concentration of the cytotoxic compound. The suitability of the proposed bioassay was demonstrated with 4-nitroquinoline 1-oxide and N-methyl-N’-nitro-N-nitrosoguanidine, obtaining LODs of 8.1 × 10^−3^ µg mL^−1^ and 0.085 μg mL^−1^, respectively [72]. Recently, Lovinskaya et al. showed a combination of three different BL biosensors for complex environmental monitoring [73]. Genotoxic, oxidant, and general toxicity were evaluated with *E. coli* strains genetically engineered to express lux operon under the control of promoters pRecA and pColD for the response to DNA-damaging agents, pSoxS and pKatG for oxidative stress, and pXen7-lux for toxicity. Increasing levels of BL emissions were observed in response to DNA damage on the biosensors pRecA-lux and pColD-lux, while decreased levels of BL were observed with the pXen7-lux biosensor. For oxidative detection, the authors reported that biosensor pKatG-lux was able to detect oxidative stress by monitoring the presence of hydrogen peroxide in the medium, while the biosensor pSoxS-lux detected superoxide anions. 

The applicability of this combined BL method was verified with river water samples from Kazakhstan. A paper-based bioluminescent and colorimetric biosensor for mercury(II) detection and toxicity assessment via smartphone was developed. The proposed biosensor was based on three biorecognition principles: a purified β-galactosidase (β-gal) enzyme which is inhibited by mercury and other metal ions, a mercury-specific *E. coli* genetically engineered to express the NanoLuc luciferase as the reporter protein, and the BL *Aliivibrio fischeri* strain to quantitatively assess the sample toxicity. The inclusion of an internal toxicity control to correct the analytical signal allowed the analysis of complex water samples providing a LOD of 0.58 ± 0.07 ppb for Hg(II) in only 60 min [40].

**Figure 3:**
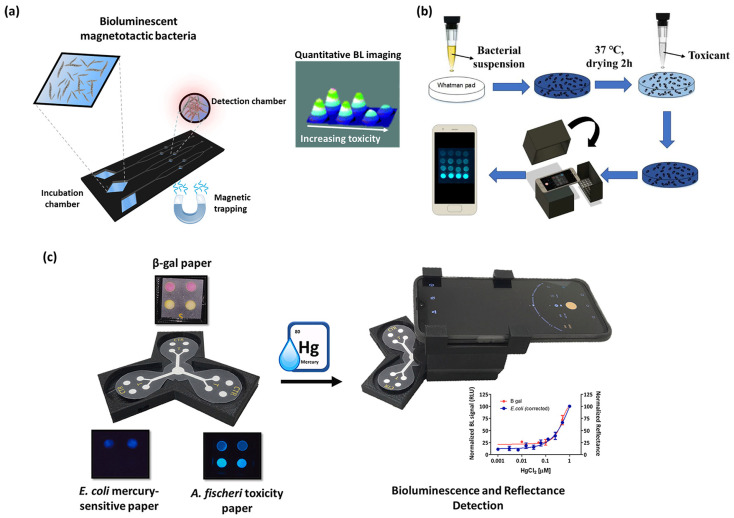
(**a**) BL magnetotactic bacteria integrated into a microfluidic chip for the evaluation of sample toxicity, reproduced from Ref. [74] with permission from the Royal Society of Chemistry. (**b**) Smartphone-based BL whole-cell biosensor for monitoring of water toxicants, reproduced from Ref. [79]. (**c**) Orthogonal paper-based biosensor for mercury (II) combining BL and colorimetric detection, reproduced from [40] with permission.

An interesting approach was previously reported in which magnetotactic bioluminescent bacteria were integrated into a microfluidic analytical device and used to assess water toxicity. This system combined the advantages of easy cell manipulation provided by magnetic properties with sensitive BL detection, with the potential to analyze very complex environmental matrices [74] (Figure 3a). 

Bacterial lux biosensors were exploited by Abilev et al. for genotoxicological studies. In particular, *E. coli* MG1655 strains were used to express the lux operon from *P. luminescens* under the control of inducible promoter genes for evaluating both the oxidative and DNA-damaging activity of potential genotoxic compounds. The applicability of the use of these BL-based biosensors for rapid screening of antioxidant and radioprotective activities was confirmed by the analysis of 29 antioxidants and radioprotectors [75]. 

Recently, a multiplexed assay based on BL 3D spherical microtissues was developed for monitoring the presence of heavy metals and for evaluating the inflammatory, antioxidant, and toxic activities of environmental samples. The assay was successfully applied to the analysis of superficial and transitional water samples, allowing us to obtain measurements of four bioactivities in a single assay, albeit still based on mammalian cell models grown in laboratory settings equipped with cell culture facilities [76].

To address full portability, microbial sensors are the best choice, and several devices have been reported in the past relying on bacterial bioreporters integrated into handheld or fully transportable equipment. However, most of these systems rely on genetically engineered organisms and, according to the regulations of several countries, they cannot be used outside authorized laboratories [77,78].

### 3.3. BL Tools for Monitoring Bioremediation Efficiency

Human activities generate toxic waste and several pollutants, affecting the environment at different levels. For example, organic and inorganic waste products are the main groundwater contaminants in oil terminal areas or industrial areas. To reduce the contamination of groundwater and water natural sources, remediation treatments are needed to avoid the spreading of these pollutants in the environment [80,81]. Thanks to the availability of rapid analytic techniques, countermeasures can be adopted when necessary [82,83]. Particular attention is devoted to alternative and convenient strategies, i.e., the use of microorganisms able to recover complex matrices to obtain the remediation of contaminated sites [84]. When exposed to organic contaminants, natural organisms can develop, by adaptation, alternative strategies to degrade potential contaminants. An example is represented by bacteria and fungi able to degrade hydrocarbons, using them as a carbon source, and a wide range of bacterial mixtures are currently available on the market for organic pollutant degradations. Additionally, biological tests are used for monitoring [82] and bioluminescent bacteria are widely exploited and commercially available for monitoring the presence of toxic compounds. Since BL intensity is directly proportional to the bacteria’s viability, the higher the degree of toxicity of the sample, the lower the amount of the BL signals emitted by bacteria [85]. Three different bioluminescent bacteria strains were employed for evaluating the bioremediation of hydrocarbon-contaminated waters and soil samples [86]. Laboratory-scale remediation experiments were performed measuring the hydrocarbon-degrading power of a culture enriched with autochthonous bacteria. These results were compared with those obtained with a commercial mixture of bacteria able to selectively degrade hydrocarbons and polychlorinated biphenyl compounds. The authors reported that the native bacteria isolated from polluted samples were less effective than commercial bacteria, probably due to the short time for selection in remediation activity despite their satisfactory capacity to degrade long-chain hydrocarbons. 

Sajayan et al. demonstrated the ability of a novel bioflocculant polysaccharide produced by *Bacillus cereus* in the bioremediation of heavy metals in wastewater by using a *Vibrio* BL reporter assay [87]. To this end, silver nanoparticles with antibacterial activities were synthetized from the polysaccharide bioflocculant and used for the bioremediation of heavy metals. By measuring the inhibition of BL expression in *Vibrio harvey* bacteria, the bioflocculant ability to remediate heavy metal toxicity was evaluated, confirming the suitability of this BL reporter assay for monitoring and testing new bioremediation strategies aimed at removing heavy metals from wastewater. 

These results suggested that the bioflocculant polysaccharide MSI021 could be used to develop more environmentally friendly wastewater treatment systems. Sutar et al. developed a method for the biodegradation and detoxification of malachite green by a newly isolated bioluminescent bacterium strain [88]. Malachite green (MG) is a dye usually used in the textile industry as a coloring agent for papers, toys, and plastic varieties. It is also used as a potential agent for treating fungal and protozoal infections in aquaculture and fisheries. The isolated bioluminescent bacterium identified as *Photobacterium leiognathi* strain MS was involved in MG dye degradation and could tolerate high concentrations of MG (1.0 g L^−1^) with 92.50% decolorization potency within 24 h. The biodegradation of MG into several metabolites as well as its catabolism pathway was confirmed by instrumental techniques such as UV-Vis, FTIR, and LC-MS QTOF analyses. One of the main bottlenecks for real-life applications still remains the scale-up of proof-of-concept methodologies. To address this issue, Mansouri et al. proposed a suitable set of methods to evaluate the efficiency of two biotreatments at a medium scale for complex sediments [89]. A total of five bioluminescent reporters were used to assess the acute toxicity of the bioremediation processes. The authors demonstrated that bioprocess efficiency differed between bioaugmented and bio-stimulated treatments in terms of organic carbon and benzo(a)pyrene (BaP) consumption and dichlorodiphenyltrichloroethane (DDT) removal performance. Organic carbon consumption was higher for the bio-enhanced treatment and directly correlated with microbial enrichment. This observation was shown to agree with the literature, where an increase in organic carbon removal of over 30% was already found with bio-enhancement approaches [90]. Furthermore, it was shown that for the two contaminants BaP and DDT, the bio-increase led to a 23% decrease in BaP compared to the bio-stimulated treatment.

One of the most promising and innovative approaches was reported by the group of Pimchai Chaiyen, who developed a method for synthesizing D-luciferin and detecting pesticides via an enzymatic cascade. For the first time, 5′-methyl-D-LH_2_, which emits a higher flux of photons with longer kinetics than D-LH_2_, was synthetized and a new technology was proposed for on-site measurement of organophosphate pesticides such as parathion, methyl parathion, profenofos [91].

Several efforts have been aimed at immobilizing BL whole-cell biosensors for point-of-need applications. For example, Ma et al. developed an easy and low-cost immobilization procedure to immobilize BL bioreporter bacteria on a filter membrane disk (Figure 3b). Different surface materials (polyester and parafilm) enriched with glucose and ampicillin were explored to improve the stability and to preserve the responsiveness of the BL bioreporter to water toxicants monitoring, allowing storage at −20 °C for three weeks. Ethanol, H_2_O_2_, and chloroform were used as model toxic compounds and BL measurements were performed with a smartphone camera, reaching LODs of 1% *v*/*v*, 0.02% *v*/*v*, and 0.0006% *v*/*v*, respectively [79].

## 4. Outlook

Improved water management is not only fundamental for achieving SDG 6 but it is also a key enabler for many other, if not all, SDGs, and the availability of green cost-effective tools is vital for ensuring water security and allowing water reuse. BL is surely a valuable tool in the arsenal of water quality monitoring tools and devices geared at providing sustainable water management solutions. Thanks to the possibility of miniaturization and the use of green reagents, it is now possible to design biosensors capable of detecting contaminants and pollutants in water systems with high accuracy. BL biosensors can facilitate early detection of water contaminations, enabling prompt intervention measures to mitigate risks to the environment and human health. However, several challenges, including technological and regulatory issues, need to be addressed before the implementation of these proof-of-principle devices to the everyday needs of the water sector. One of the main issues affecting the accuracy of BL cell biosensors is related to nonspecific effects and interferences of sample matrix on the BL signal, causing under- or, less frequently, over-estimations of the concentration of target analyte. Therefore, a viability control should be always introduced, either as an internal control, i.e., a reporter protein constitutively expressed in the same cell, or as a separate viability control strain.

Microbial fuel cells and lab-on-a-chip biosensors for water quality monitoring are becoming increasingly popular. A wide range of enzymatic biosensors, both free and coupled with nanomaterials, immunosensors, cell-free transcription translation biosensors, or luminescent biosensors have been developed to detect specific targets such as pathogens, and different water contaminants with great accuracy. They have also been implemented in portable devices to develop systems for rapid and on-site detection and monitoring. In addition, biosensors can be integrated into wireless sensor networks to monitor the environment and they have the potential for resource recovery, supporting a circular economy. They can act as tools for the detection of new resources that can be extracted from wastewater and contribute to carbon neutrality. However, further research is needed to develop and scale up these technologies for practical implementation. Leveraging them for resource recovery in addition to monitoring can enhance their economic viability and environmental benefits.
